# Standardizing Antimicrobial Use in a Resource-Limited Pediatric Surgical Unit in Botswana

**DOI:** 10.1093/ofid/ofag083

**Published:** 2026-03-19

**Authors:** Alemayehu Ginbo Bedada, Mazvita Rankin, Andrew P Steenhoff, Eimear Kitt

**Affiliations:** Department of Surgery, Faculty of Medicine, University of Botswana, Gaborone, Botswana; Global Health Center, Children's Hospital of Philadelphia, Philadelphia, Pennsylvania, USA; Global Health Center, Children's Hospital of Philadelphia, Philadelphia, Pennsylvania, USA; Department of Paediatric and Adolescent Health, Faculty of Medicine, University of Botswana, Gaborone, Botswana; Department of Pediatrics, Perelman School of Medicine, University of Pennsylvania, Philadelphia, Pennsylvania, USA; Botswana–UPenn Partnership, Gaborone, Botswana; Global Health Center, Children's Hospital of Philadelphia, Philadelphia, Pennsylvania, USA; Department of Pediatrics, Perelman School of Medicine, University of Pennsylvania, Philadelphia, Pennsylvania, USA; Botswana–UPenn Partnership, Gaborone, Botswana; Department of General Paediatrics, Children's Health Ireland, Dublin, Ireland; Department of Infectious Diseases, Children's Health Ireland, Dublin, Ireland

**Keywords:** Africa, AWaRe, clinical pathways, pediatric surgery, stewardship

## Abstract

**Background:**

Antimicrobial resistance is rampant in low- and middle-income countries. Recent data from Princess Marina Hospital (PMH), Botswana, revealed that 100% of pediatric surgical unit patients received antimicrobials inappropriately.

**Methods:**

We implemented a quality improvement initiative to improve antimicrobial use in children admitted to PMH's pediatric surgical ward. With key stakeholders, we developed clinical pathways (CPs) to standardize antimicrobial use across common surgical diagnoses. A CP booklet, informed by the World Health Organization (WHO) Access, Watch, and Reserve (AWaRe) guideline, was distributed to prescribers. We conducted weekly prospective antimicrobial use audits over 1 year, from 3 months pre–CP implementation to 9 months post–CP implementation.

**Results:**

A total of 1099 pediatric surgical patients were admitted and 374 (34.0%) required antimicrobials. The WHO Access group accounted for 360 antibiotic courses (72.4%) and the Watch group for 137 (27.6%), a total of 497. Overall, appropriate antimicrobial use improved significantly (pediatric surgery, 33 [50.8%] vs 99 [93.4%]; orthopedics, 3 [10.3%] vs 26 [89.7%]; neurosurgery, 5 [27.8%] vs 13 [72.2%]; and ear, nose, and throat, 4 [33.3%] vs 19 [95.0%]; each *P* < .001) in the postimplementation period except for maxillofacial-dental patients (1 [25.0%] vs 3 [75.0%]; *P* = .264). Improvements were observed across provider categories and years of experience: medical officers (28 [42.4%] vs 38 [91.0%]), interns (7 [33.3%] vs 20 [87.0%]), and specialists (11 [26.2%] vs 64 [97.0%]); years of experience: <2 years (9 [32.1%] vs 22 [91.7%]), 2–5 years (3 [25.0%] vs 50 [92.6%]), and >5 years (34 [38.2%] vs 154 [92.2%]) (*P* < .001 for each).

**Conclusions:**

Appropriate antimicrobial use improved post–CP implementation. Expanding CPs with ongoing antimicrobial stewardship education will ensure sustained improvement.

## Teaser

Antimicrobial use was overall low on the surgical ward, with 34.0% of admitted surgical patients receiving an antimicrobial at least once. Appropriate use of antimicrobials improved after implementation of clinical pathways. Improvement in prescribing correlated with level of providers’ experience.

Antimicrobials are critical tools in reducing the global burden of morbidity and mortality caused by bacterial infections [1–4]. However, inappropriate use is abundant, ranging from 50% to 85% [5, 6], contributing to antimicrobial resistance and adverse clinical outcomes [1, 7–10]. Low- and middle-income countries (LMICs) have been identified by the World Health Organization (WHO) as being disproportionately affected by antimicrobial resistance [11, 12], posing a major global health threat [7] that requires urgent mitigation [2, 13, 14].

Antimicrobial stewardship (AMS) is designed to improve the appropriate use of antimicrobials and patient outcomes [3, 8, 13, 15, 16]. The necessary infrastructure to support AMS is often lacking in LMICs, where needs are highest [17]. In Botswana, the emergence of antimicrobial resistance is well described [18–20]. This includes pediatric patients where a high rate of inappropriate antimicrobial use in the pediatric surgical ward was reported at Princess Marina Hospital (PMH) [21].

Here, we describe the implementation of a quality improvement intervention designed to standardize the use of antimicrobials on the pediatric surgical ward using clinical pathways (CPs) to guide appropriate antimicrobial prescribing. We compared the proportion of appropriate prescribing pre– and post–CP implementation and assessed proportions of use of WHO Access, Watch, and Reserve (AWaRe) classes of antimicrobials. We also explored the appropriateness of prescribing among providers by different levels of experience. Though guidelines are a well-described tool to assist in improving patient outcomes, to our knowledge, this is the first description of the impact of locally developed guidelines on antimicrobial prescribing in an LMIC pediatric surgical setting.

## METHODS

### Setting and Participants

We conducted a quality improvement initiative over a 1-year period, from 17 September 2023 to 16 September 2024, in the PMH pediatric surgical unit. PMH is the largest (530-bed) tertiary care hospital in Botswana and is located in Gaborone, the capital city. Admission records were monitored for the study period and all patients receiving antimicrobials were invited to participate in the study.

The official bed capacity of the pediatric surgical ward (PSW) is 30, although the number of admitted patients may, at times, be higher. All pediatric surgical subspecialty patients, including those undergoing emergency and elective procedures, were admitted to the ward including general pediatric surgery, orthopedics, neurosurgery, otolaryngology, maxillofacial-dental (MFD), and ophthalmology. Surgical specialist staffing was provided by 2 pediatric surgeons, 2 orthopedic surgeons, 4 neurosurgeons, 2 otolaryngologists, 5 MFD surgeons, and 1 ophthalmologist. Typically, children aged between 28 days and <13 years were admitted to the PSW; however, in special circumstances, patients up to 15 years of age were admitted.

### Study Design and Data Collection

Data collection took place prospectively over a 12-month period, including a 3-month pre–CP implementation (pre-CP) period and a 9-month post–CP implementation (post-CP) period. During the pre-CP period, key stakeholders (surgical specialists and 2 pediatric infectious disease specialists) contributed to the development of 13 CPs for the treatment of common surgical pathologies that required antimicrobial treatment. These included acute appendicitis, burn sepsis and toxic shock syndrome, cellulitis, pediatric acute intestinal obstruction, postneurosurgical procedure meningitis, skull fractures, ventriculoperitoneal shunt infections, deep neck infections, tonsilitis, fractures, hand infection, osteomyelitis, and septic arthritis (Supplementary Material, “Clinical Pathways”). Data were collected using REDCap and included demographic details, admitting surgical specialty, level of prescriber's experience (categorized by <2 years, 2–5 years, or >5 years), and clinical outcomes. We also captured antibiotic data with antimicrobial use documented on admission, and daily thereafter, the choice of the antimicrobial agent, dose, and route and confirmation whether it was given or not. Based on this, we captured if it was appropriate or not. Additional variables included pain medication prescribing and drug shortages (Supplementary Table A). Notably, before this project, there were no established CPs on the PSW. Interns rotated through the department for only 8 weeks. Educational sessions on the developed CPs for prescribers and nurses were initiated at the beginning of the post-CP period and revisited every 3 months, with progress reports provided during regular morning meetings. Routine surgical prophylaxis was prescribed at the time of surgery and was excluded from the data. As a control variable, we also compared the proportion of patients receiving pain medication in the pre-CP and post-CP periods to assess external factors affecting prescribing practices.

### Outcomes

The primary outcome of the study was the proportion of patients receiving appropriate antimicrobials for the infectious diagnosis in question. We defined appropriate antimicrobial use as the correct choice and dose of agent as outlined in the CP for the clinical scenario in question. If there was no documented indication for the prescribed antibiotic, it was counted as inappropriate. Our secondary outcomes included comparison of the WHO AWaRe categories of antimicrobials prescribed in the pre-CP and post-CP periods. The WHO AWaRe classification is a system that categorizes antimicrobials into 3 groups (Access, Watch, and Reserve) to guide their appropriate use. The Access class of antimicrobials should be consistently available serving as first-choice and occasionally second-choice antimicrobials with low toxicity and used for common infections [2, 10, 22]. The Watch class of antimicrobials comprises critically important antimicrobials prescribed for specific infections with a relatively higher toxicity and resistance potential compared to the Access class of antimicrobials [10, 22]. The Reserve class of antimicrobials is used as a last-resort choice where Access or Watch classes cannot be used, for example, in treatment of multidrug-resistant bacteria [2, 10, 22]. Last, we compared antimicrobial prescribing practices according to the level of the prescribing physician, in the pre-CP and post-CP periods (intern, medical officer, or specialist) and also by years of practice (<2 years, 2–5 years, or >5 years). We also noted appropriateness of antimicrobial prescribing according to length of stay (<5 days, 5–10 days, >10 days), and we explored drug shortages as a resource limitation that may have contributed to inappropriate prescribing practices.

### Data Analysis

The data were checked for completeness and exported to IBM SPSS software version 30 for analysis. Continuous variables were described using median (interquartile range [IQR]). Categorical variables were described using proportions and compared using the χ^2^ or Fisher exact test, as appropriate. Descriptive statistics of the population were completed, along with daily and overall proportions of antimicrobial use. The pre-CP baseline data were compared with the post-CP data to determine the impact of the developed CPs on appropriate prescription of antimicrobials. The appropriateness of antimicrobials in relation to the level and years of experience of the prescribers during the pre-CP and post-CP periods was determined and compared. A *P* value <.05 was considered statistically significant.

### Ethical Statement

This study was approved by the institutional review boards (IRBs) of the University of Botswana, the Botswana Ministry of Health, and PMH. Written informed consent was obtained from all guardians, and assent was provided by children aged >7 years, following a thorough explanation of the research objectives, benefits, options for withdrawal at any time, and the minimal or no risks associated with participation. Consent and assent were requested by a bilingual study assistant in either Setswana or English according to participant or guardian preference.

## RESULTS

### Sociodemographic Description

All patients admitted during the study period were approached to participate. No patients declined to give consent, and none were lost to follow-up. A total of 1099 pediatric surgical patients were admitted during the 1-year study period. Of these, 374 required antimicrobials at least once (34.0%). The proportion of patients who received 2 or more antibiotics during the post-CP period (41/245 [16.7%]) was significantly lower than during the pre-CP period (41/129 [31.8%]) (*P* < .001). Of these, 119 (31.8%) were female and 255 (68.2%) were male. The overall median age was 5.1 (IQR, 1.8–9.5) years.

**Figure 1.**
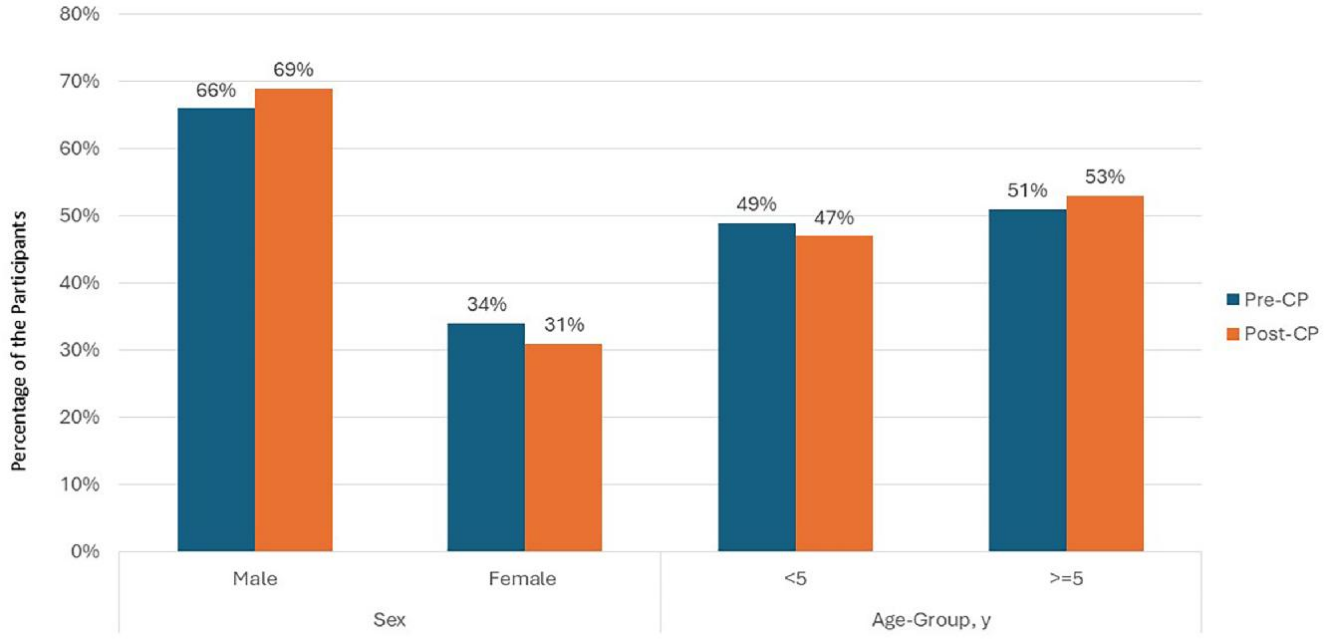
Sex and age distribution of pediatric surgical admissions in the 3-month pre-CP vs 9-month post-CP periods of the study in the pediatric surgical ward of Princess Marina Hospital. Abbreviations: Post-CP, post–clinical pathway implementation; Pre-CP, pre–clinical pathway implementation.

Demographics of patients receiving antimicrobials were similar between patients in the pre-CP and post-CP periods. In the pre-CP period, 34.1% of the cohort was female (n = 44), and the median age was 5.0 (IQR, 1.5–9.4) (0.08–16.5) years. During the post-CP period (9 months), 30.6% of the cohort was female (n = 75) and the median age was 5.3 (IQR, 1.8–9.5) (0.05–17.5) years. The proportions of patients aged <5 years and ≥5 years were similar (*P* = .784), and there was no significant difference in sex distribution (*P* = .490) (Figure 1).

Across both pre-CP and post-CP periods, general pediatric surgery admission was the most common category for admission at 50.4% and 43.3%, respectively, followed by orthopedic admissions at 22.5% and 31.0%, respectively (Table 1). Supplementary Table B shows the diagnoses during the pre-CP and post-CP periods.

**Table 1.** Surgical Admission by Specialty and Age

Specialty	Pre-CP Period	Post-CP Period	Age <13 y	Age ≥13 y
Pediatric surgery	65 (50.4%)	106 (43.3%)	162	9
Orthopedics	29 (22.5%)	76 (31.0%)	104	1
Neurosurgery	18 (14.0%)	25 (10.2%)	42	1
Ear, nose, and throat	12 (9.3%)	20 (8.2%)	32	0
Maxillofacial-dental	4 (3.1%)	18 (7.3%)	21	1
Ophthalmology	1 (0.8%)	0 (0.0%)	1	0
Total	129 (100.0%)	245 (100.0%)	362 (96.8%)	12 (3.2%)

Abbreviation: CP, clinical pathway implementation.

### Antimicrobial Utilization

Among the 17 types of antimicrobials used, 16 were classified under the WHO AWaRe framework: 10 were Access and 6 were Watch classes of antimicrobials. Amphotericin was the additional agent used, which is not classified in the framework. These 17 antimicrobials were used in a total of 498 counts over the study period. Of these, 37 (17 pre-CP and 20 post-CP) were documented at admission, 21 (56.8%) administered intravenously (IV) and 16 (43.2%) by mouth (PO). After admission, 461 counts were used: 264 (57.3%) IV, 195 (42.3%) PO, and 2 (0.4%) intramuscular. Overall, IV antimicrobials were used in 285 of 498 (57.2%), with 118 of 190 (62.1%) during the pre-CP period and 167 of 308 (54.2%) in the post-CP period (*P* = .093).

Of the 497 counts of antibiotic use, 359 (72.2%) were categorized as Access and 138 (27.8%) as Watch. On admission, the use of the Access and Watch groups of antimicrobials in the pre-CP (13/17 [76.5%] vs 17/20 [85.0%]) and post-CP (4/17 [23.5%] vs 3/20 [15.0%]) periods, respectively, was similar (*P* = .680). After admission, a total of 460 AWaRe class of antimicrobials were administered, with the use of Access class of antimicrobials increasing in the post-CP period compared with the pre-CP period (217/287 [75.6%] vs 112/173 [64.7%], respectively; *P* < .001) (Supplementary Table C).

The 3 most commonly used antimicrobials during pre-CP and post-CP periods were similar: amoxicillin-clavulanate (52/190 [27.4%] vs 121/308 [39.5%]), metronidazole (35/190 [18.4%] vs 45/308 [14.6%]), and cefotaxime (37/190 [19.5%] vs 34/308 [11.0%]), respectively (Supplementary Table D).

### Appropriateness of Antimicrobial Utilization

**Figure 2.**
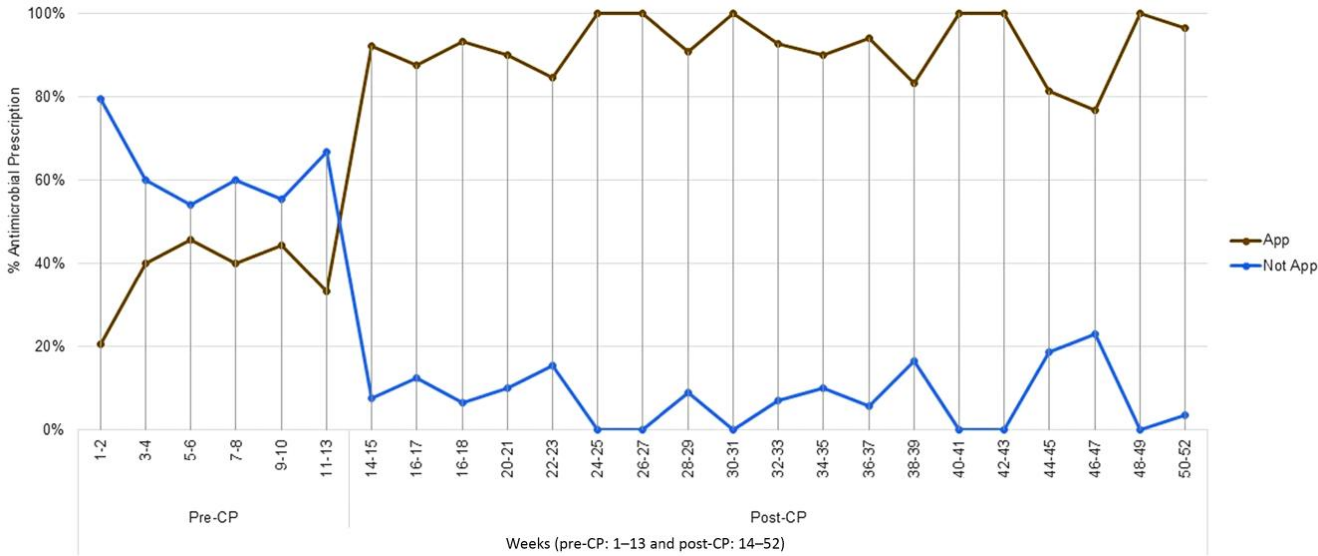
The trend in appropriate antimicrobial use over the study period is shown biweekly. Abbreviations: App, appropriate; Post-CP, post–clinical pathway implementation; Pre-CP, pre–clinical pathway implementation.

The average proportion of patients prescribed antimicrobials daily was 6.8%: 7.6% during the pre-CP period and 6.5% during the post-CP period (*P* = .337). In the pre-CP period, 11 patients were on antibiotics at admission, of whom 5 (45.5%) had an appropriate prescription, while after admission, 129 patients received antibiotics, with 46 (35.7%) prescribed appropriately. During the post-CP period, 14 patients were on antibiotics at admission, of whom 13 (92.9%) had an appropriate prescription, while after admission, 245 patients received antibiotics, with 226 (92.2%) prescribed appropriately (Figure 2 and Table 2).

**Table 2.** Appropriate Use of Antimicrobials According to Specialty in the Pre– and Post–Clinical Pathway Implementation Periods

Specialty	Appropriate Antimicrobial Use^a^	Pre-CP Period	Post-CP Period	*P* Value
Antimicrobial use at admission	Yes	5 (45.5%)	13 (92.9%)	.**021**
	No	6 (54.5%)	1 (7.1%)	
Antimicrobials after admission	Yes	46 (35.7%)	226 (92.2%)	**<.001**
	No	83 (64.3%)	19 (7.8%)	
Pediatric surgery	Yes	33 (50.8%)	99 (93.4%)	**<.001**
	No	32 (49.2%)	7 (6.6%)	
Orthopedics	Yes	3 (10.3%)	26 (89.7%)	**<.001**
	No	73 (96.1%)	3 (3.9%)	
Neurosurgery	Yes	5 (27.8%)	13 (72.2%)	**<.001**
	No	23 (92.0%)	2 (8.0%)	
Ear, nose, and throat	Yes	4 (33.3%)	19 (95.0%)	**<.001**
	No	8 (66.7%)	1 (5.0%)	
Maxillofacial-dental	Yes	1 (25.0%)	3 (75.0%)	.264
	No	12 (66.7%)	6 (33.3%)	

Abbreviation: CP, clinical pathway implementation.

*P*-values in bold indicate statistically significant results.

^a^Appropriate = overall/all prescribed antimicrobials to each patient is/are appropriate.

At admission, in the pre-CP period, 13 Access and 4 Watch group of antimicrobials were prescribed: 7 (53.8%) and 3 (75.0%) were appropriate, respectively. After admission, in the pre-CP period, 111 Access and 62 Watch group of antimicrobials were prescribed: 47 (42.3%) and 26 (41.9%), respectively, were appropriate.

At admission, in the post-CP period, 17 Access and 3 Watch group of antimicrobials were prescribed: 16 (94.1%) and 3 (100.0%), respectively, were appropriate. After admission, in the post-CP period, 204 Access and 83 Watch group of antimicrobials were prescribed: 188 (92.2%) and 80 (96.4%), respectively, were appropriate (Supplementary Table E).

Our control variable of pain medications use throughout the study period showed that 117 (90.7%) of patients received pain medication in the pre-CP period compared to 224 (91.4%) in the post-CP period (*P* = .813).

### Prescriber Experience and Antimicrobial Utilization

During the pre-CP period, there were no statistically significant differences in prescription appropriateness among prescribers based on their years of experience and positions of the prescribers (Supplementary Table F). Post-CP, professionals at all roles and levels of experience demonstrated a statistically significant improvement in the rate of appropriate antimicrobial prescription (*P* < .001 for each group; Table 3).

**Table 3.** Prescribers, Their Experience Level, and Antimicrobial Use

Experience Level	Period	Appropriate Antimicrobial Use^a^	Nonappropriate Antimicrobial Use	*P* Value
Medical officer	Pre-CP	28 (42.4%)	38 (57.6%)	**<.001**
	Post-CP	38 (91.0%)	14 (9.0%)	
Intern	Pre-CP	7 (33.3%)	14 (66.7%)	**<.001**
	Post-CP	20 (87.0%)	3 (13.0%)	
Specialist	Pre-CP	11 (26.2%)	31 (73.8%)	**<.001**
	Post-CP	64 (97.0%)	2 (3.0%)	
<2 y	Pre-CP	9 (32.1%)	19 (67.9%)	**<.001**
	Post-CP	22 (91.7%)	2 (8.3%)	
2–5 y	Pre-CP	3 (25.0%)	9 (75.0%)	**<.001**
	Post-CP	50 (92.6%)	4 (7.4%)	
>5 y	Pre-CP	34 (38.2%)	55 (61.8%)	**<.001**
	Post-CP	154 (92.2%)	13 (7.8%)	

Abbreviation: CP, clinical pathway implementation.

*P*-values in bold indicate statistically significant results.

^a^Appropriate = overall/all prescribed antimicrobials to each patient is/are appropriate.

### Length of Hospital Stay and Drug Shortages

Of the patients receiving antimicrobials, most 153 (40.9%) were admitted for >10 days. One hundred seventeen (31.3%) patients had a hospital stay of ≤5 days, and 104 (27.8%) patients had a 6- to 10-day hospital stay. There was no significant statistical difference in the appropriate use of antimicrobials among those with a hospital stay of <5 days versus 6–10 days (*P* = .717), ≤5 days versus >10 days (*P* = .398), and 6–10 days versus >10 days (*P* = .664) (Supplementary Table G).

Antimicrobial shortage was formally reported only once in the study period, when amphotericin B was unavailable for a *Candida guilliermondii* ventriculo-peritoneal shunt infection. However, antimicrobial shortages were later reported to be significantly higher during a qualitative study at the same time [23]. The majority of patients receiving antimicrobials 358 (95.2%) were discharged home well. However, 2 patients (0.5%) died during the study period, 1 from sequelae of sepsis and the other due to a postoperative complication. Fourteen patients (3.7%) were referred to other health institutions for further treatment.

## DISCUSSION

The overall proportion of patients who received therapeutic antimicrobials in this PSW in Gaborone, Botswana, at least once was 34%, which is overall low for surgical specialties. Our study demonstrated that the overall average daily proportion of patients on antimicrobials was 6.8%. There was a reduction from 7.6% during the pre-CP period to 6.5% during the post-CP period (not statistically significant).

The prevalence of in-hospital use of antimicrobials in sub-Saharan hospitals ranges from 36.8% to 80.6% [11, 12, 24], and 82.9% in the United States [25]. A study from Botswana reported a 70.6% point prevalence of antimicrobials use across many hospitals [26]. The significantly lower proportion of patients receiving antimicrobials in our cohort may be partially due to the fact that we only included pediatric patients, as compared to other studies that included adults too, but we feel it is likely that there was also a greater awareness of antimicrobial stewardship given the recent development and implementation of our CP program.

Our study showed a dramatic improvement in antimicrobial prescribing throughout the study period. Overall, appropriate antibiotic use increased from 36% to 92% with orthopedics in particular seeing the largest rise in appropriate prescribing. Even MFD, while not statistically significant, experienced an increase in appropriate prescribing. The modest MFD improvement could be partly because of their limited participation in multiple feedback sessions in general surgical morning report as the MFD team conduct their morning meetings separately. A study from the same population in PMH 5 years prior reported a 100% inappropriate use of antimicrobials in the same PSW where this study was conducted and a 52% rate of inappropriate use in the pediatric medical ward [21]. Our results are promising for a long-lasting and sustainable improvement in practice, given that preexisting improvements were already being noted. The significant improvement observed in our study may be attributed to the CPs that were developed mainly by local stakeholders. Previous research has shown that treatment guidelines tailored to common pathologies and based on local data can promote adherence [13]. Our control variable of pain medication use throughout the study period showed no significant change in pain medication prescribing, further supporting this theory.

Our secondary outcome was to compare the potential changes in WHO AWaRe categories being prescribed. We noted a significant increase in the proportion of use of the Access class of antimicrobials during the post-CP period of our study. The overall use of Access and Watch classes of antimicrobials was 69.4% and 30.6%, respectively. By comparison, a study from the pediatric ward of the same hospital reported a 42.0% use of Access class and a 58.0% of Watch class of antimicrobials [21]. In other studies, the use of the Access class of antimicrobials was reported to increase from 47.4% to 74.8% and Watch class from 23.7% to 38.4% [24, 27]. In our study, no Reserve class of antimicrobials was used. This was similarly reported in a study that involved many sub-Saharan countries [21, 27].

The 3 most common antimicrobials used in our study were amoxicillin-clavulanate, metronidazole, and cefotaxime. This is consistent with published literature in similar but not identical settings where they mainly used the Access and Watch class of antibiotics regionally and globally [5, 11, 12, 24, 26–28].

In a recent systematic review of point-prevalence studies on antibiotics use among hospitalized patients in Africa, amoxicillin-clavulanate was among the most commonly used antibiotics [29]. A recent point-prevalence study involving children in Kenya revealed it to be the most commonly prescribed antibiotic at one of the sites [30]. Another point-prevalence study from Ghana reported amoxicillin-clavulanate to be one of the most common antibiotics prescribed in pediatrics, as well as one of the top antibiotics used for surgical prophylaxis. While this study did not assess the appropriateness of antibiotic use, the suspicion of unnecessary double anaerobic cover was raised [23].

Our study showed a significant and enhanced improvement in appropriate prescription of antimicrobials in all prescribers and all levels of experience. The quality of antimicrobial prescribing is influenced by multiple factors, including hospital and cultural contexts, patient-specific characteristics, and the experience of prescribers [31]. A study from Benin found that 54.8% of pharmacy staff lacked knowledge of the definition of antimicrobial therapy [32]. In our setting, senior prescribers were more familiar with the CPs and likely contributed to their development. They would also be expected to have a robust baseline knowledge base to improve from and a greater awareness of the sociocultural environment of PMH and may also have a greater commitment to appropriate antibiotic use compared with junior staff such as interns, who often rotate through the PSW for limited time periods. This is reflected in the higher rate of appropriate prescriptions during the post-CP period among specialists (97.0%), compared to medical officers (91.0%) and medical officer interns (87.0%). However, the difference among these providers group were not statistically significant, likely due to substantial improvements across all 3 categories.

The mortality was similar to a mortality rate of 0.8% reported from the United States in a study that aimed to identify antimicrobial stewardship targeting pediatric surgical patients [25].

The shortage of antimicrobials was officially reported once. However, in a qualitative study in the same cohort [33], shortage of antimicrobials was more commonly reported when specifically asked. None of our patients were reported as missing a dose of antimicrobials in their stay. This is an improvement from the 15 patients who were reported as missing their doses in a report from the pediatrics medical and surgical wards of the same hospital in 2018 [21]. This could be partly due to higher rate of usage of Access group of antimicrobials, which are listed in the national essential drug list.

The increased use of Access-class antimicrobials and the reduction in multiple-antibiotic prescribing practices underscore the potential of CPs to align clinical practice with global antimicrobial stewardship goals. Sustaining the gains observed after CP implementation requires a multifaceted strategy, including ongoing education and mentorship and integration of CPs into institutional policies. In resource-constrained settings, low-cost interventions such as peer review, standardized surveillance, and multidisciplinary stewardship teams are essential to ensuring long-term impact. Furthermore, embedding CPs within national antimicrobial stewardship frameworks is critical for sustaining and scaling these improvements hospital-wide.

### Limitations

This study was conducted in PMH, the largest pediatric surgical center in the country, which serves more than half of the pediatric population of Botswana. With multiple pediatric subspecialty teams and an affiliated medical school, PMH has robust research infrastructure that we believe greatly enhanced the implementation of our study. Despite this, we acknowledge this infrastructure may not be present in all LMIC settings.

The limitations of our study include its exclusively quantitative design. In addition, the dataset was constrained by the absence of information on the total duration of antibiotic use, microbiological data that could inform the appropriateness of antibiotic selection, and standardized reporting of drug shortages. Although common clinical diagnoses were selected, the number of pathways developed was limited and did not encapsulate all admissions. Furthermore, care providers’ levels of training were broadly categorized (specialists, residents, medical officers, and nurses), and years of experience were captured in intervals, which precluded more granular analyses, including assessment of year-to-year differences.

## CONCLUSIONS

Antimicrobial prescribing improved significantly post–CP implementation of CPs in this LMIC setting. The use of Access class of antimicrobials increased during the post-CP period. Healthcare providers across all levels of training and experience demonstrated improved appropriateness in antimicrobial prescribing. Furthermore, the prescription of >1 antibiotic for a single patient was significantly reduced. We recommend further detailed study on the full appropriateness of antimicrobial utilization including the right antimicrobials, right dose, right route, and right duration for the right condition to optimize clinical outcomes with minimal unintentional consequences of antimicrobials and expansion of clinical pathways with ongoing antimicrobial stewardship education to sustain our observed improvements.

## Supplementary Material

ofag083_Supplementary_Data

## Supplementary Data

Supplementary materials are available at *Open Forum Infectious Diseases* online. Consisting of data provided by the authors to benefit the reader, the posted materials are not copyedited and are the sole responsibility of the authors, so questions or comments should be addressed to the corresponding author.
