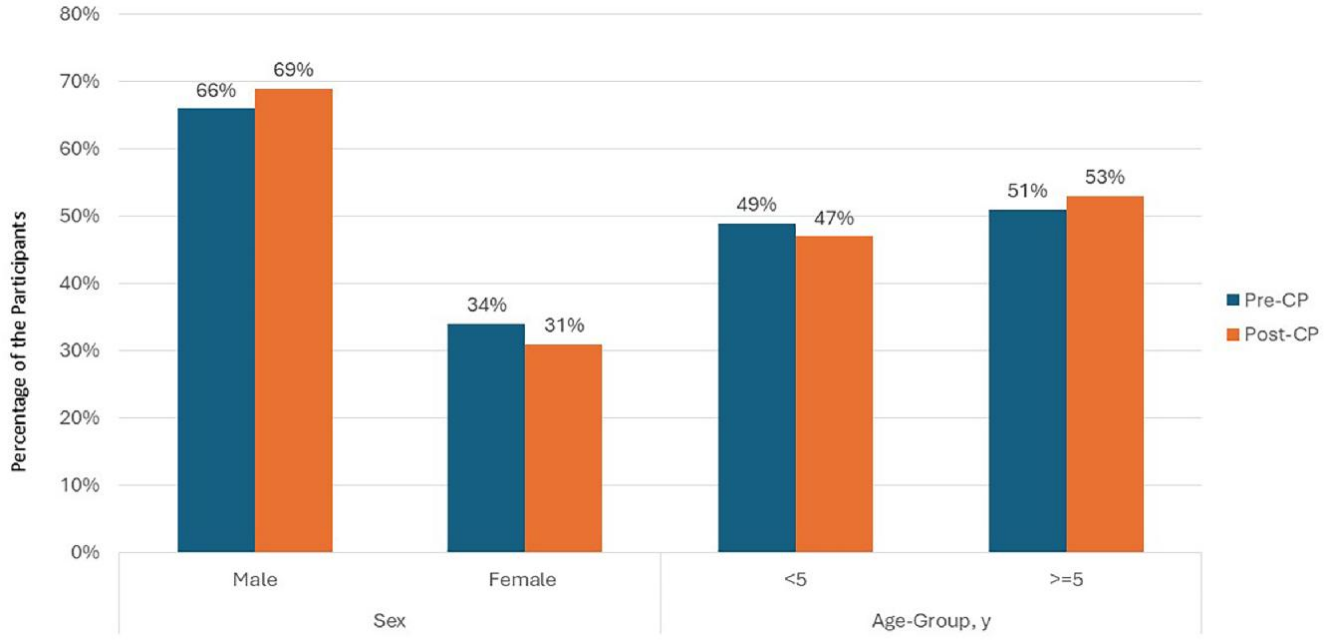# Correction to: Standardizing Antimicrobial Use in a Resource-Limited Pediatric Surgical Unit in Botswana

**DOI:** 10.1093/ofid/ofag470

**Published:** 2026-08-01

This is a correction to: Alemayehu Ginbo Bedada, Mazvita Rankin, Andrew P Steenhoff, Eimear Kitt, Standardizing Antimicrobial Use in a Resource-Limited Pediatric Surgical Unit in Botswana, *Open Forum Infectious Diseases*, Volume 13, Issue 3, March 2026, ofag083, https://doi.org/10.1093/ofid/ofag083

In the originally published version of this manuscript, the percentage for age 5 and above in the Pre-CP category was incorrectly reported as 27% within Figure 1.

The correct value is 51% and Figure 1 has been amended accordingly.

The corrected Figure 1 reads:

**Figure:**