# Supplementary material for: Standardizing Antimicrobial Use in a Resource-Limited Pediatric Surgical Unit in Botswana
Source: Open Forum Infect Dis. 2026 Mar 19;13(3):ofag083. doi: 10.1093/ofid/ofag083 (PMC13000887; doi:10.1093/ofid/ofag083)
Supplement: ofag083_Supplementary_Data [file ofag083_supplementary_data.zip › Supplementary Material_ CPS.docx]

**Princess Marina Hospital and the University of Botswana Paediatric Surgical Clinical Pathways**


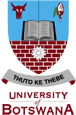

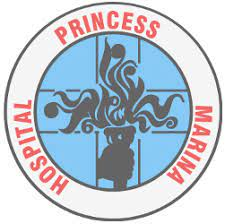


December 2023

Table of Content

| Team | Topics | Page |
| --- | --- | --- |
| Paediatric surgery | Acute Appendicitis | 3-7 |
|  | Burn sepsis and toxic shock syndrome | 8-11 |
|  | Cellulitis | 12-14 |
|  | Paediatric acute intestinal obstruction | 15-16 |
| Neurosurgery | Post-neurosurgical procedure meningitis | 17 |
|  | Skull fractures | 17 |
|  | VP shunt infections | 18-19 |
| ENT | Deep Neck Infection | 20-21 |
|  | Tonsilitis | 21-22 |
| Orthopaedics | Fractures | 23 |
|  | Hand infection | 24-27 |
|  | Osteomyelitis | 27-28 |
|  | Septic arthritis | 28-29 |
| References | | 30 |

**ACUTE APPENDICITIS**

***Clinical Presentation***

- Pertinent Symptoms
  - Poorly localized abdominal pain that usually localizes to the RLQ
  - Anorexia (universal), nausea, emesis, fever, diarrhoea
- Pertinent Signs/Physical Exam Findings
  - Guarding, localized RLQ tenderness, percussion tenderness
  - Rovsing, obturator or psoas sign may be found.
  - Diffuse peritonitis → free appendix perforation

***Treatment algorithm based on clinical suspicion***

- HIGH
  - Immediate appendectomy
- INTERMEDIATE
  - US- study of choice/Re-examine.
    - If Positive → appendectomy
    - If Uncertain → admit/re-examine
    - If Negative → discharge or alternative diagnosis → treatment
- LOW
  - Reliable → Discharge with advice/follow-up in 24hrs

***Diagnosis***

- Lab studies
  - FBC, U and E, urinalysis (routine and microscopic or dip-stick)
    - Pertinent findings: possible leukocytosis (not always), urinalysis most negative for infection
- Radiographic Studies
  - Ultrasound
    - Findings → Identification of a tubular, non-compressible, aperistaltic bowel loop showing a connection between the cecum and a distal blind end (appendix) with a diameter of > 6mm. A calcified appendicolith may appear as a curved, echogenic structure with posterior acoustic shadowing.
    - Non-Perforated vs. Perforated
      - Non-Perforated → > 6mm, fluid-filled, local peri-cecal and pelvic fluid, appendicolith
      - Perforated → > 6mm, increased free intraperitoneal fluid (localized or generalized), increased peri-appendiceal echogenicity.
      - Abscess

***Management***

- **NON-PERFORATED APPENDICITIS**
  - Pre-Operative Management
    - IV fluids/NPO
    - Analgesia
    - Antibiotics
      - 1^st^ line: Augmentin - initial choice of antibiotics. If not available, can use ampicillin + gentamicin + metronidazole.
      - If the patient has a significant penicillin allergy, initiate ciprofloxacin + metronidazole.
      - Single dose at the time of diagnosis; continue further if indicated.
- Definitive treatment
  - Immediate appendectomy
- Post-operative Management
  - Immediately post-op
    - NPO, IV fluids, Analgesia
    - NO Antibiotics (in uncomplicated appendectomy)
  - 24-48hrs post-op → Advance diet to clear fluids
  - 48hrs post-op → Advance diet as tolerated to a regular diet.
    - Stop IV fluids.
    - Discharge home with pain medication
  - 2 weeks post-op
    - Return to the clinic for wound check and post-operative assessment.
- **PERFORATED APPENDICITIS (NO ABSCESS)**
- Pre-Operative Management
  - NPO, IV fluids: IV fluids/NPO – 10 - 20 mL/Kg NS or LR
- Analgesia
- Antibiotics
  - - 1^st^ line: Augmentin - initial choice of antibiotics. If not available, can use ampicillin + gentamicin + metronidazole.
    - If the patient has a significant penicillin allergy, initiate ciprofloxacin + metronidazole.
- Definitive management
  - - Resuscitation → Immediate appendectomy
- Post-operative Management
  - Immediately Post-op: NPO, IV fluids
    - Continue IV Antibiotics
    - 1^st^ line: Augmentin - initial choice of antibiotics. If not available, can use ampicillin + gentamicin + metronidazole.
      - If the patient has a significant penicillin allergy, initiate ciprofloxacin + metronidazole.
    - 2^nd^ line drug if no clinical response in 72 hrs or clinical deterioration – Piperacillin + tazobactam
    - 3^rd^ line drug – meropenem + vancomycin and consider ID consultation.
  - 24-48hrs post-op → Advance diet to clear fluids
    - Continue IV Antibiotics
  - 48hrs post-op → Advance diet as tolerated to a regular diet.
    - Stop IV fluids.
    - Convert to PO antibiotics when culture sensitivities are back; will need a 7-DAY COURSE of antibiotics post-op.
    - Discharge home with antibiotics and pain medication when tolerating a regular diet.
  - 2 weeks post-op
    - Return to the clinic for wound check and post-operative assessment.
- **PERFORATED APPENDICITIS (ABSCESS versus PHELGMON)**
- ABSCESS > 4-6cm
  - Initial Orders → NPO, IV fluids
  - Antibiotics
- 1^st^ line: Augmentin or ceftriaxone + metronidazole or cefotaxime + metronidazole
- 2^nd^ line: if no clinical response in 72 hrs or clinical deterioration: Piperacillin + tazobactam
- 3^rd^ line: ID consult and consider meropenem + vancomycin.
- Appendectomy +/- Drainage
  - - Laparotomy or laparoscopy for washout or percutaneous US-guided.
    - Send pus for microscopy and culture, give directed antibiotics once sensitivity results are out.
- Improvement → Regular diet, Oral Antibiotics (consult ID specialist for the total duration) and discharge.
- PHLEGMON/ABSCESS < 4cm
  - Fever or Sepsis
    - Follow the pathway of abscess > 4 cm.
  - Afebrile
    - Regular diet, Oral Antibiotics, and discharge.

| **Antibiotics** | **IV dose** | **PO dose** |
| --- | --- | --- |
| Before prescribing, be sure to check the dose in a formulary, check for drug-drug side effects and ensure the drug is safe for your patient. | | |
| Ceftriaxone | 50-100/kg/day IV every 24 hours (max 2000 mg) |  |
| Metronidazole | 10 mg/kg/dose IV every 8 hours (max 2000 mg) | 7.5 mg/kg/dose PO QD (Max/dose: 500 mg) |
| Ciprofloxacin | 10 mg/kg/dose IV every 8 hours (Max/dose: 400 mg; max/day: 1200mg) | 20 mg/kg/dose PO BD (Max/dose: 750 mg; max/day 1500 mg) |
| **Piperacillin/Tazobactam** | 100 mg/kg/dose every 8 hours (Max 3000 mg) |  |
| **Cefotaxime** | 50-180 mg/kg /day divided into 8 hourly (max 2 gm |  |
| **Augmentin** | 30mg/kg/dose every 8 hours (max 1.2g) | Augmentin 10 mg/kg every 8 hours (dosage above 10 mg of clavulanic acid will cause diarrhoea)  Then add Amoxycillin 15 mg/kg every 8 hours |
| **Clindamycin** | 20-40 mg/kg/day divided into 8 hourly doses (max 600 mg) | 20-40 mg/kg/day divided into 8 hourly doses (max 600 mg) |
| **Amikacin** | Loading dose 25 mg/kg, then 15 mg/kg every 24 hrs(daily) |  |
| **Meropenem** | 20 mg/kg every 8 hours (max 1g 8 hourly) |  |
| **Vancomycin** | 10 mg/kg/dose every 6 hours |  |

**BURN SEPSIS AND TOXIC SHOCK SYNDROME**

***Clinical Presentation***

- Pertinent symptoms
  - Fever, irritability, lethargy
  - Decreased PO intake, vomiting, diarrhoea.
- Pertinent signs/Physical Exam findings
  - Look for the source of the infection.
  - Fever >38.9
  - Persistent and progressive Tachycardia
  - Tachypnoea
  - hypotension
  - Evidence of ileus or abdominal distention
  - Confusion/ depressed level of consciousness

***Treatment algorithm based on clinical level of suspicion.***

***Diagnosis***

- Clinical
  - It can be difficult to differentiate SIRS and sepsis, failure to differentiate SIRS from sepsis results in poor antibiotic stewardship and failure to recognize sepsis results in septic shock, multi-organ failure and ultimately death
  - SIRS: 2 or more of the following
    - Temperature >38^0^C or <36^o^C
    - Heart rate >2SD for age or Bradycardia of <10^th^ percentile
    - Respiratory rate >2SD for age
    - WCC leucocyte count elevated or depressed for age or >10% immature neutrophils
  - Sepsis: At least 3 of the following
    - Temperature >38^0^C or <36^o^C
    - Progressive tachycardia >2SD for age
    - Progressive tachypnoea > 2 SD for age
    - Refractory hypotension < 2 SD for age
    - Leucocytosis >12 000 cells/μl or <4 000 cells/μ l
    - Thrombocytopenia <100 000 cells/μl
    - Hyperglycaemia > 6.1 mmol/l
    - Inability to tolerate feeds > 24 hrs
- Laboratory
  - Full blood count- white blood count and cell differential, Haemoglobin, platelets
  - Urea and electrolytes, CMP (calcium, magnesium, and phosphate)
  - Blood culture
  - Wound swab
  - Procalcitonin (PCT), Urine Microscopy and Culture
  - Coagulation profile (for DIC)
- Imaging (US, CT, MRT, etc.)
  - Chest X-ray

***Treatment***

- Wound Washing
  - Early wound care is important in the reduction of burn infection as well as associated morbidity and mortality.
  - Initial management involves cleaning the wound with chlorhexidine soap and removing devitalised tissue.
  - Infection rates are significantly reduced if the burn is washed within 8 hours, advise referring facility to wash and remove all blisters before transfer.
  - The wash should include the entire patient including hair, fingers, and toes. Shave hair to 2 cm clear of burn on the scalp.
  - Ensure adequate analgesia has been given, use warm water and ward the room to avoid hypothermia.
  - Rinse and dress the patient with available dressing; options are silver sulfadiazine, chloramphenicol ointment/tetracycline ointment with Vaseline gauze, Bactigras (chlorhexidine impregnated paraffin gauze) for the face.
- Do not administer routine prophylactic antibiotics.
  - If criteria for sepsis are met, then treat with antibiotics.
  - Sepsis at day 1 to 3 of burn
    - Intravenous antibiotics; Cloxacillin 50 mg/kg 6hrly, Gentamicin 6mg/kg daily, Ampicillin 50 mg/kg 6hrly
    - Burn in the first 48 hrs post-burn is a high risk for Staph/Strep toxic shock syndrome.
  - Sepsis at day 4 of burn or more (where concern is now higher for Pseudomonas infection)
    - Piptazobactam 50mg/kg 6hrly and Amikacin loading dose 25 mg/kg, then 18mg/kg daily
    - Review antibiotics once culture and sensitivities are available.
- Toxic Shock Syndrome
  - Toxic shock syndrome (TSS) is a toxin-mediated infection (streptococcus pyogenes or staphylococcus aureus) and is the most common cause of mortality in small burns.
  - It is typically seen in children 1-4 years within 72 hrs of burn and can result in significant morbidity and mortality of up to 50% if untreated.
  - Disease is characterised by hypotension, fever, erythema, maculopapular rash, lethargy, confusion, irritability, diarrhoea and vomiting.
- Laboratory – thrombocytopenia, lymphopenia, drop in Hb by >4%, hyponatremia.
  - Any child with a fever of > 38.9^0^C, a rash, or a sudden change in clinical condition within a few days of a burn injury should be monitored closely for TSS.

***Management***

- Once there is a clinical suspicion of TSS, insert IV access and start the patient on resuscitation Intravenous fluid.
- Transfer patient to high care bed with a monitor, urgent referral to ICU. Do not leave the patient unattended.
- Collect blood for FBC, U&E, CMP, Crossmatch, blood culture, coagulation profile and blood gas.
- Give intravenous fluid bolus (10ml/kg of 0.9 Normal Saline) and start inotropes.
- Initiate antibiotics as per above guideline
- In addition, stop toxin, and cytokine production with clindamycin 15-25mg/kg 8 hrly
- If no clinical improvement after 6 hours, give intravenous immunoglobulin IVIG at 2g/kg, if not available or clinically unstable administer 10ml/kg FFP
- Review patient hourly until there is clinical improvement.
- Consider theatre dressing change.

Age-specific vital signs

| Age | Tachycardia/  Bradycardia | Hyperthermia/  hypothermia | Systolic blood pressure | Leukocytosis | Leukopenia | Respiratory rate |
| --- | --- | --- | --- | --- | --- | --- |
| 0 – 1 week | >180/<100 | >38.5/<36 | <65 | >34 | N/A | >50 |
| 1 week – 1 month | >180/<100 | >38.5/<36 | <75 | >19.5 | <6 | >40 |
| 1 month – 1 year | >180/<90 | >38.5/<36 | <100 | 17.5 | <6 | >34 |
| 2 – 5 years | >140 | >38.5/<36 | <94 | 15.5 | <6 | >22 |
| 6 – 12 years | >130 | >38.5/<36 | <104 | 13.5 | <4.5 | >18 |
| 13- 18 years | >110 | >38.5/<36 | <117 | >11 | <4.5 | >14 |

**CELLULITIS**

***Definitions***

- Cellulitis non-purulent: infection involving the deep dermis and subcutaneous tissue
  - Microbes: streptococci (group a most common, others b, c, f or g) and s. aureus - Penetrating wounds
- Cutaneous abscess - purulent: collection of pus in the dermis and deeper tissues
  - Microbe: S. Aureus – could be MRSA (although seems less common in Botswana)
- Necrotizing fasciitis: aggressive subcutaneous infection that tracks along the superficial fascia – wooden-hard induration of the subcutaneous tissue.
  - Microbes: S. Pyogenes, others (S. Aureus, V. Vulnificus, A. Hyrdrophila, anaerobic streptococci)

***Clinical Presentation***

- Pertinent symptoms
  - Abrasion, bites (insect, animal), primary dermatologic conditions
  - Fever, history of pustule/abscess, drainage
- Pertinent signs/physical exam findings
  - Erythema, rash, induration, swelling, fluctuation
  - Regional lymphadenopathy

***Diagnosis***

- Healthy children with uncomplicated cellulitis/abscess: routine lab not recommended.
- Children with significant systemic symptoms/sirs
  - Lab studies
    - FBC - possible leucocytosis (not always), U&E, CRP, blood glucose, LFT’s if indicated.
    - Culture: blood, wound, others as indicated.
  - Radiographic studies
    - Ultrasound – abscess:
      - Hypoechoic area with thickening of the surrounding subcutaneous tissue as compared to the normal surrounding tissue. Irregular borders are often seen in early stages of formation, they progress to more well-circumscribed borders seen in later stages.
      - Pus “swirling” around within the abscess cavity (squish sign): apply gentle pressure with the probe and then release.
    - Ultrasound – cellulitis:
      - The image has the appearance of “cobble stoning” with the oedema (hypoechoic) tracking within the septations of the subcutaneous tissue.
    - X-ray of affected limb- to assess for evidence of osteitis or osteomyelitis, refer to orthopaedics as necessary.

***Management***

- Non-purulent cellulitis (s. Pyogenes – most likely)
  - Analgesia
  - Inpatient antibiotics - iv
    - 1^st^ line: iv Cefazolin: 20 mg/kg/dose TD (max/dose: 1 gm) or Cloxacillin 50mg/kg/dose QID (max 2g)
    - 2^nd^ line or penicillin allergy iv
      - Clindamycin 10-20 mg mg/kg/dose TD (max/dose: 600 mg)
      - Vancomycin 10 mg/kg/dose for MRSA sepsis 5-7 days duration (but 14 days if bacteraemic)
  - Outpatient antibiotics – patients without significant systemic symptoms - oral
    - 1^st^ line: cephalexin: 50 mg/kg/day divided td (max/dose: 500 mg) – 5 days.
    - 2^nd^ line or penicillin allergy clindamycin: 10 -20 mg/kg/dose td (max/day: 600 mg)
  - Not improving in > 48 hours: consider the possibility of abscess, empiric antibiotics change.
- Cutaneous abscess with or without cellulitis > 2 cm – (s. Aureus – most likely)
  - Incision and drainage
  - Inpatient
    - Cefazolin: 20 m/kg/dose td (max/dose: 1 gm) or Cloxacillin 25-50mg/kg/dose max 2g
    - or clindamycin as 2^nd^ line or antibiotic allergy 10- 20 mg/kg/day divided into 8 hourly doses (max 600mg)
    - Vancomycin 10 mg/kg/dose for MRSA sepsis 5-7 days duration
    - Typical duration is 5 days from clinical improvement or drainage. If MRSA bacteremia or sepsis, consider longer (10-14 days)
  - Outpatient – po
    - Cephalexin: 50 mg/kg/day divided td (max/dose: 500 mg) or
    - clindamycin 10- 20 mg/kg/day divided into 8 hourly doses (max 600mg)
    - Typical duration is 5 days from clinical improvement or drainage. If MRSA bacteremia or sepsis, consider longer (10-14 days)
  - Not improving in > 48 hours: consider need for us, I&D or repeat I&D, empiric antibiotics or ID consult.
- Cutaneous abscess single < 2 cm
- Incision and drainage
- Patients with systemic symptoms, concern with inadequate drainage, multiple sites, immunodeficiency or younger age
  - Cefazolin: 20 mg/kg/dose td (max/dose: 1 gm) or Cloxacillin
  - or clindamycin as 2^nd^ line or antibiotic allergy 10- 20 mg/kg/day divided into 8 hourly doses (max 600mg)
  - Vancomycin 10mg/kg/dose for MRSA sepsis 5-7 days duration. Typical duration is 5 days from clinical improvement or drainage. If MRSA bacteraemia or sepsis, consider longer (10-14 days)
- Not improving in > 48 hours: consider the possibility of abscess, empiric antibiotics change.
- Necrotizing fasciitis
- Surgical debridement until viable tissue is reached.
- Amikacin + piperacillin-tazobactam
- Add Vancomycin for MRSA sepsis.

**PAEDIATRIC ACUTE INTESTINAL OBSTRUCTION**

***Clinical presentation***

- Pertinent symptoms
  - Patient should have a history of abdominal surgery.
  - Nausea and vomiting
  - Abdominal distention and pain
  - No recent passage of stool or flatus
- Pertinent signs/Physical Exam findings
  - Abdominal distention
  - Abdominal tenderness
  - fever
  - Peritonism
  - Haemodynamic instability due to hypovolemia or sepsis
  - Evidence of dehydration e.g. sunken eyes, reduced urine output

***Treatment algorithm based on clinical level of suspicion.***

***Diagnosis***

- Laboratory
  - Full blood count with cell differential
  - Urea and electrolytes, Calcium, Magnesium and Phosphate
  - Blood gas
- Imaging (US, CT, MRT, etc.)
  - Abdominal X-ray
  - Small bowel or colonic distention with air-fluid levels
  - Pneumoperitoneum

***Treatment***

- Preoperative
  - Administer nothing per mouth, NPO.
  - Insert an intravenous cannula and initiate fluid resuscitation with either 0.9 Normal Saline or Ringers Lactate.
  - Correct electrolyte imbalance
  - Insert a nasogastric tube and leave on free drainage.
  - Replace nasogastric tube loss at a ratio of 1:1.
  - Initiate patient of maintenance fluids using dextrose-containing fluids to avoid hyperglycaemia.
  - Provide adequate analgesia.
  - It is not necessary to initiate the patient on prophylactic antibiotics.
  - Administer broad-spectrum antibiotics only if bowel necrosis or perforation is suspected.
    - 1^st^ choice: Co-Amoxycillin clavulanate intravenous 25 mg/kg/day in divided doses
    - 2^nd^ choice: Cefotaxime 150 mg/kg/day in divided doses, used in combination with metronidazole 15mg/kg/day in divided doses.
- If there is a resolution of intestinal function, the patient can be discharged home.
- Criteria for Surgical Intervention
- Peritonism
- Worsening abdominal pain
- Fever
- Tachycardia
- Localised or generalised tenderness.
- Haemodynamic instability
- Pneumoperitoneum in Abdominal x-ray.
- On-table preoperative antibiotics
- Cefazolin 30 mg/kg, maximum of 2 g per dose.
- Metronidazole 12.5 mg/kg up to 500 mg per dose.
- Should be given within 30-60 minutes of initial incision.
- Repeat dose after 4 hours if surgery is delayed or prolonged.
- Prophylaxis doses are given to reduce the risk of surgical site infection.
- Postoperative
- Keep Patient NPO with a nasogastric tube on free drainage.
- Reinitiate feeds once gut function resolves (bilious aspirates clear from NGT)
- Continue IV maintenance with dextrose-containing fluid.
- Ensure adequate analgesia is prescribed.
- Continue antibiotics as treatment if intra-abdominal soiling or contamination.
- Otherwise continue prophylactic antibiotics for 24- 48 hours
  - 1^st^ choice: Co-Amoxycillin clavulanate intravenous 25 mg/kg/day in divided doses.
  - 2^nd^ choice: Cefotaxime 150 mg/kg/day in divided doses, used in combination with metronidazole 15 mg/kg/day in divided doses.

**POST-NEUROSURGICAL PROCEDURE MENINGITIS**

***Clinical Presentation***

- Pertinent symptoms
  - Surgical procedure on the brain
- Pertinent signs
  - Vital signs
  - Signs and symptoms of meningitis.

***Diagnosis***

- Usual organisms: Coagulase-negative staphylococci, S. aureus, Enterobacteriaceae, Pseudomonas spp., Strep pneumoniae
- Culture and sensitivity

***Treatment***

- Empiric
  - Vancomycin and cefepime first line, then ciprofloxacin/vancomycin for penicillin-allergic
  - Second line or resistant Gram-negative infection - meropenem

**SKULL FRACTURES**

***Clinical presentation***

- Pertinent symptoms
  - History of trauma
- Pertinent signs/physical examination
  - Vital signs
  - Neurologic and associated injuries

***Diagnosis***

- Imaging: CT scan

***Treatment***

- Basal skull fracture (BSF)
  - Prophylactic – routine use is not recommended.
  - BSF Traversing nasal sinuses: Ciprofloxacin for 7 – 10 days OR Cephalosporin

**VP SHUNT INFECTION**

***Clinical Presentation***

- Pertinent Symptoms
  - Headache, vomiting or poor feeding.
  - Weakness, sleepy,
- Pertinent Signs/Physical examination
  - Fever, Cushing’s Triad, decreased breath sounds.
  - Fontanelle bulging, increased head circumference.
  - Pain, redness, swelling at shunt site, over tubing.
  - Abdominal tenderness, ? increase in abdominal girth
  - Upward gaze paresis, abnormal gait, change in spasticity.

***Diagnosis***

- Lab:
  - FBC, CRP, U&E, CSF cytochemical analysis – before antibiotics
  - Blood culture
- Imaging
  - Limited shunt radiography: Single AP or PA radiograph of chest and abdomen
  - Abdominal Ultrasound: to evaluate for pseudocyst
  - Brain CT: Ultra low-dose and scout lateral radiograph to include entire neck to clavicles

***Treatment***

- First-line antibiotics
  - Cefepime, IV: (if not available, can use ceftriaxone or cefotaxime: use formulary to give high dose as for meningitis)
    - < 50 kg: 50 mg/kg/dose every 8 hours, Maximum: 2,000 mg/dose
    - > 50 kg and/or > 18 years: 2000 mg TD

AND

- Vancomycin, IV:
  - < 50 kg: 15 mg/kg/dose every 6 hours, Maximum: 750 mg/dose> >
  - > 50 kg and/or > 18 years: 15 mg/kg/dose every 6 hours, Maximum: 1000 mg/dose
- Second-line Antibiotics for severe penicillin or cephalosporin allergy
- Ciprofloxacin, IV:
  - < 40 kg: 10 mg/kg/dose every 8 hours, Maximum: 400 mg/dose
  - > 40 kg: 400 mg every 8 hours.

AND

- Vancomycin, IV:
  - < 50 kg: 15 mg/kg/dose every 6 hours, Maximum: 750 mg/dose
  - > 50 kg and/or > 18 years: 15 mg/kg/dose every 8 hours, Maximum: 1000 mg/dose

Note:

- Give cefepime, ciprofloxacin, or meropenem BEFORE vancomycin.
- If CSF Gram stain positive for Gram-negative rods, change therapy to meropenem AND vancomycin and suggest ID consult.
  - Meropenem, IV
    - - - ≥ 1 month and < 50 kg - 40 mg/kg/dose every 8 hours, Maximum: 2,000 mg/dose
        - ≥ 50 kg and/or ≥ 18 years; 2,000 mg every 8 hours

**DEEP NECK INFECTIONS**

**(Retropharyngeal abscess, Lateral pharyngeal abscess, and parapharyngeal abscess)**

***Clinical Presentation***

- Pertinent symptoms
  - Fever, pain with swallowing, difficulty of swallowing, poor oral intake, change in voice.
  - Decreased ability to move the neck (especially extension) for pain
- Pertinent signs/physical examination
  - Drooling,
  - Respiratory distress, tachypnoea, stridor, trismus

***Diagnosis***

- Lab:
  - FBC, blood culture, CRP
- Imaging:
  - Lateral neck x-ray:
    - A prevertebral space of < 6 mm at the level of C3 is considered normal in children. Alternatively, in general, in infants between 2 and 5 years of age, the **retropharyngeal** soft tissues (C1-C3) should be < ½ the AP length of C5 vertebral body, and the **retrotracheal** soft tissues (C4-C7) should be < the AP length of C5 vertebral body. In paediatric patients, widening of the prevertebral soft tissues can be a normal spurious finding that is related to expiration, crying, and/or suboptimal neck extension.​​
  - Minimize unnecessary computed tomography (CT) imaging in patients who do not need surgical intervention as part of treatment for deep neck space infections.

***Treatment***

- First-line treatment
  - Less severe disease
    - - No MRSA risk factor: Ampicillin/Sulbactam, IV: 50 mg/kg/dose QD, Max 2000 mg ampicillin component/dose.
      - MRSA risk present: Clindamycin IV: 14 mg/ka/dose ID, Max 900 mg /dose
  - Severe disease (? airway compromise)
    - Ampicillin/sulbactam, IV: 50 mg/kg/dose QD, Max 2000 mg ampicillin component/dose AND Vancomycin IV: < 50 kg – 15 mg/kg/dose QD, Max 750 mg/dose; > 50 kg and/or >18 years: 15 mg/kg/dose TD, Max 1000 mg/dose
- Second-line treatment (penicillin allergy)
- Less severe disease
  - - No MRSA risk factor: Clindamycin, IV: 14 mg/kg/dose TD, Max 900 mg/dose
- Severe disease
  - - Ceftriaxone, IV: 100 mg/kg/dose BD, Max 2000 mg/dose AND Vancomycin, IV: < 50 kg: 15 mg/kg/dose QD, Max 750 mg/dose; > 50 kg and/or > 18 years: 15 mg/kg/dose TD, Max 1000 mg/dose AND Metronidazole, IV: Infant: PMA > 44 weeks, Children and adolescents: 7.5 mg/kg/dose QD, Max 500 mg/dose

**TONSILLITIS**

***Clinical presentation***

- Pertinent symptoms
  - Sore throat, fever, headache
  - Other symptoms include dysphonia, odynophagia, halitosis, and snoring for the paediatric population (not as an acute infection, but rather due to the size of adenoids, and tonsils) and can also cause feeding difficulties in children.
- Pertinent signs
  - Inflamed/erythematous tonsillar mucosa with congestion (often viral), with exudates (bacterial tonsillitis)

***Diagnosis***

- Laboratory
  - FBC, ESR, CRP (inflammatory markers), baseline (RFT, LFT)
  - Throat swab for MCS
  - Complicated by Peritonsillar Abscess (needle aspiration and MCS, REFER for Incision and drainage) or retropharyngeal abscess.
- Imaging (US, CT, MRT, etc.)
  - Nil imaging recommended for uncomplicated tonsillitis,
  - If a deep space neck infection, then consider:
  - Lateral neck X-ray for prompt diagnosis.
    - Consider CT neck with contrast where necessary.

***Treatment***

- Preoperative
  - Good hand hygiene and infection prevention
  - NPO, IV fluids, Analgesia
  - Antibiotics
    - 1^st^ choice: Augmentin
    - 2^nd^ choice: Cephalosporins (cefpodoxime, cefuroxime) oral if tolerated, IV ceftriaxone, or cefotaxime and metronidazole.
- Operative treatment- tonsillectomy
  - Indications:
  - Recurrent infection: 3 or more episodes in the past 3 years; more than 7 well-documented clinically treated episodes in the preceding year. Or 5 or more for the preceding 2 years, Or peritonsillar abscess, 2 weeks after incision and drainage of Quinsy
  - Suspected malignancy (such as in unilateral tonsillar swelling)
  - Halitosis from debris in the tonsillar crypts (“Tonsil stones”)
  - Obstructive Sleep Apnoea Syndrome.
- Postoperative
  - Analgesia
  - IVF if significant odynophagia – give TFI (reassess and switch to orals once tolerated),
  - Soft diet, oral fluids
  - Antibiotics
  - 1^st^ choice: nil recommended.
  - 2^nd^ choice: secondary post-operative infections treat as pre-operatively.
- On Discharge
  - Antibiotics
    - 1^st^ choice: nil recommended (complete course started for secondary infection) usually 5 days.

**FRACTURES** – open

***Clinical presentation***

- Pertinent symptoms
  - History of trauma
- Pertinent signs/physical examination
  - Vital signs
  - Bone fractures and associated injury.

***Diagnosis***

- Clinical
- X-rays

***Treatment***

- Type I: blunt or penetrating - < 1 cm and clean
  - Frist-line Agent: Cefazolin 2 gm IV TD
  - Second-line agent; Clindamycin 900 mg IV QD
- Type II: blunt or penetrating - 1-10 cm without significant tissue damage/involvement
  - First and second-line agents similar to Type I
- Type III: Blunt or penetrating - 10 cm, segmental fracture, extensive soft tissue damage, traumatic amputation, femoral fracture.
  - First-line Agent
  - Ceftriaxone 2 gm IV OD
  - Second-line agent
  - Clindamycin 900 mg IV TD AND Gentamycin 5 mg/kg IV/dose
- With Contamination – fracture-related crush or vascular injury, faecal contamination, standing water, soil.
- First-line Agent
  - Ceftriaxone 2 gm IV OD AND
  - Metronidazole 500 mg IV TD
- Second-line agent
  - Clindamycin 900 mg IV TD AND Gentamycin 5 mg/kg IV/dose

**HAND INFECTION**

**Deep infection of the hand**

- Involve the subfascial spaces of the hand and forearm: Thenar, Hypothenar, Midpalm, Interdigital space, dorsal sub aponeurotic space of the hand, and Parona’s space of the volar forearm.
- The most common organisms: S. aureus and Streptococcus spp.
- Most follow a penetrating injury.
- Interdigital (Web) space Infection

***Clinical presentation***

- Pertinent symptoms
  - Break in the skin between the fingers, penetrating trauma
  - Painful swelling of distal palmar region
- Pertinent signs/physical examination
  - Abducted adjacent finger – large collection.

***Diagnosis***

- Clinical

***Treatment***

- Surgical incision and drainage
- Antibiotics
  - First line: Augmentin
- Penicillin allergy and complex infection: Ciprofloxacin + Clindamycin
  - Second line: Clindamycin

**Dorsal subcutaneous and sub aponeurotic space**

***Clinical presentation***

- Pertinent symptoms
  - penetrating trauma
  - Painful swelling of distal palmar region and marked oedema
- Pertinent signs/physical examination
  - Erythema, tenderness

***Diagnosis***

- Clinical

***Treatment***

- Elevation, splint immobilization
- Surgical drainage – fluctuation
- Antibiotics
  - First line: Augmentin
- Penicillin allergy and complex infection: Ciprofloxacin + Clindamycin
  - Second line: Clindamycin

**Thenar space infection**

***Clinical presentation***

- Pertinent symptoms
  - penetrating trauma
  - Painful active or passive movement,
- Pertinent signs/physical examination
  - Oedema, exquisite tenderness
  - Abducted thumb – large abscess

***Diagnosis***

- Clinical

***Treatment***

- Surgical drainage – palmar incision
- Antibiotics
  - First line: Augmentin
  - Penicillin allergy and complex infection: Ciprofloxacin + Clindamycin
  - Second line: Clindamycin

**Mid-palmar infection**

***Clinical presentation***

- Pertinent symptoms
  - penetrating trauma
  - Painful swelling of the palmar
- Pertinent signs/physical examination
  - Edema of the volar and dorsal surface of the hand
  - Effaced normal palmar concavity.
  - Tender palm, tender passive flexion and extension of the fingers

***Diagnosis***

- Clinical

***Treatment***

- Surgical drainage – fluctuation
- Antibiotics
  - First line: Augmentin
- Penicillin allergy and complex infection: Ciprofloxacin + Clindamycin
  - Second line: Clindamycin

**Hypothenar Space infection**

***Clinical presentation***

- Pertinent symptoms
  - penetrating trauma
  - Painful
- Pertinent signs/physical examination
  - Edema and tenderness of the hypothenar eminence
  - Tenderness with flexion of the small finger

***Diagnosis***

- Clinical

***Treatment***

- Surgical drainage – fluctuation
- Antibiotics
  - First line: Augmentin
  - Penicillin allergy and complex infection: Ciprofloxacin + Clindamycin
  - Second line: Clindamycin

**Parona’s space infection**

- Communicates with radial and ulnar bursa and mid-palmar space!

***Clinical presentation***

- Pertinent symptoms
  - penetrating trauma
  - Painful swelling of the distal forearm
- Pertinent signs/physical examination
- Edema, tenderness, fluctuance – volar forearm
- Tenderness on passive flexion of the wrist and fingers

***Diagnosis***

- Clinical

***Treatment***

- Surgical drainage – fluctuation
- Antibiotics
  - First line: Augmentin
  - Penicillin allergy and complex infection: Ciprofloxacin + Clindamycin
  - Second line: Clindamycin

**OSTEOMYELITIS**

***Clinical Presentation***

- Pertinent Symptoms
  - Recent infectious symptoms or URT infection, skin infection, fever, pain,
  - Poor appetite, fatigue
  - Recent trauma, surgical history
- Pertinent Signs/Physical examination
  - Fever, hypotension, tachycardia, tachypnoea.
  - Irritable, refuse to bear weight, limp.
  - Rash, trauma sign,
  - Focal erythema, tenderness to palpation or motion.

***Diagnosis***

- Lab:
  - FBC, CRP, blood culture
  - CK (myolysis): not usually sent as does not influence management.
- Imaging:
  - Plain x-ray
  - MRI (osteomyelitis suspected)

***Treatment***

- Targets – S. aureus (MSSA and MRSA), S. pyogenes (Gp-A), Kingella kingae (small bones, 6/12 – 4 years), Salmonella in patients with sickle cell disease
- First-line antibiotics
- No MRSA risk factors
  - **Cefazolin, IV:** 35 mg/kg/dose every 8 hours, Max: 2,000 mg/dose
  - **Allergy to first-**line agent: **Clindamycin, IV:** 14 mg/kg/dose every 8 hours, Max: 900 mg/dose (confirm clindamycin susceptibility)
- MRSA risk factors
  - **Clindamycin, IV:** 14 mg/kg/dose every 8 hours, Max: 900 mg/dose (confirm clindamycin susceptibility)
  - If concern for  Kingella kingae in a patient with MRSA risk factors, add **Cefazolin, IV:** 35 mg/kg/dose every 8 hours, Max: 2,000 mg/dose
- Osteomyelitis with Gram-positive bacteraemia (susceptibility pending)
  - **Vancomycin, IV**
    - Infants, children and adolescents ≤ 50 kg:15 mg/kg/dose IV every 6 hours, Max: 750 mg/dose
    - Children and adolescents > 50 kg: 15 mg/kg/dose IV every 8 hours, Max: 1,000 mg/dose

**SEPTIC ARTHRITIS**

***Clinical Presentation***

- Pertinent Symptoms
  - Pain, fever, rash, recent infectious symptoms,
  - Trauma history
- Pertinent signs/Physical Examination
- Tachycardia, fever
- Rash, wound, swelling, erythema, warmth, tenderness, limited range of movement,
- Limp, inability to bear weight.

***Diagnosis***

- Lab:
  - FBC, CRP, ESR
  - Bacterial culture and gram stain, blood culture
  - Synovial fluid studies – Gram stain, cell count, and culture
- Imaging
  - X-rays
  - US

***Treatment***

- With effusion – Joint aspiration
- Suspected Septic arthritis: Target pathogen: S. aureus, S. pyogenes (Gp-A), Kingella kingae
- First-line therapy
- **Cefazolin, IV:** 35 mg/kg/dose every 8 hours; maximum: 2,000 mg/dose
- **If MRSA risk factor(s) present:**
- **Vancomycin, IV:** 14 mg/kg/dose every 8 hours; maximum: 900 mg/dose (If the prior history of clindamycin-resistant MRSA, therapy guided by prior isolate’s susceptibility
- Second line for severe Penicillin allergy or cephalosporin allergy
- **Vancomycin if severe disease IV:** 14 mg/kg/dose every 8 hours; maximum: 900 mg/dose
- **Synovial fluid positive for gram negative organisms**
- **Ceftriaxone, IV:** 50 mg/kg/dose every 24 hours; maximum: 2,000 mg/dose

**REFERENCES**

1. Young AE, Thornton KL. Toxic shock syndrome in burns: diagnosis and management. *Archives of Disease in Childhood - Education and Practice*2007;92:ep 97-ep100
2. Pomeroy S, Young AE, Williams P. Burns (Paediatric): Toxic Shock Syndrome and Sepsis. University Hospitals Bristol NHS Foundation Trust. [www.uhbristol.nhs.uk](http://www.uhbristol.nhs.uk). Version 2 Feb 18 - Review Feb 21
3. Goldstein B, Giroir B, Randolph A, International Consensus Conference on Pediatric S International Pediatric sepsis consensus conference: definitions for sepsis and organ dysfunction in pediatrics. *Pediatric critical care medicine: a journal of the Society of Critical Care Medicine and the World Federation of Pediatric Intensive and Critical Care Societies.*2005;6(1):2–8. Epub 2005/01/08
4. Griffin K, Minneci P, Halaweish I. Adhesive small bowel obstruction clinical pathway. Nationwide Children’s hospital. NCH Evidence Based Practice Guideline Development Manual v4.1.2022
5. Surgical Antimicrobial prophylaxis Guidelines appendix 13: Paediatric surgical procedures. S*outh Australian expert Advisory Group on Antibiotic Resistance (SAAGAR). Reviewed December 2021*
