# Supplementary material for: Standardizing Antimicrobial Use in a Resource-Limited Pediatric Surgical Unit in Botswana
Source: Open Forum Infect Dis. 2026 Mar 19;13(3):ofag083. doi: 10.1093/ofid/ofag083 (PMC13000887; doi:10.1093/ofid/ofag083)
Supplement: ofag083_Supplementary_Data [file ofag083_supplementary_data.zip › Supplementary Tables A-G.docx]

**Supplementary Table A**: Variables of interest.

| **Category** | **Variable** |
| --- | --- |
| Demographics | Age and gender |
| Paediatric surgical subspeciality | Paediatric surgery, paediatric orthopaedics, neurosurgery, ENT, Maxillo-facial-dental, ophthalmology |
| Prescribers’ experience | <2 years, 2-5-years, and >5 years |
| Antimicrobials | Names of antimicrobials, dose, route, given or not, appropriateness, |
| Pain medication | Names of pain medications, dose, route, given or not |
| Limitations | Names of drugs and reasons |
| Clinical outcome | Discharged, referred, died, and length of hospital stay |

**Supplementary Table B**: Diagnoses in Pre- and Post-Clinical Pathway periods.

| **Diagnoses** | **Pre-CP** | **Post-CP** | **Total** |
| --- | --- | --- | --- |
| Acute abdomen | 2 (66.7%) | 1 (33.3%) | 3 |
| Acute scrotum | 0 (0.0%) | 5 (100.0%) | 5 |
| Adenoid hypertrophy | 5 (45.5%) | 6 (54.5%) | 11 |
| Anorectal malformations | 7 (50.0%) | 7 (50.0%) | 14 |
| Appendicitis | 12 (28.6%) | 30 (71.4%) | 42 |
| Bone tumour | 0 (0.0%) | 3 (100.0%) | 3 |
| Bone/joint deformities | 0 (0.0%) | 6 (100.0%) | 6 |
| Bowel obstruction | 9 (50.0%) | 9 (50.0%) | 18 |
| Brain tumour | 0 (0.0%) | 2 (100.0%) | 2 |
| Burns | 11 (50.0%) | 11 (50.0%) | 22 |
| Cleft lip or/and palate | 2 (16.7%) | 10 (83.3%) | 12 |
| Empyema | 1 (100.0%) | 0 (0.0%) | 1 |
| Oesophageal achalasia | 1 (50.0%) | 1 (50.0%) | 2 |
| Foreign body - GI | 3 (50.0%) | 3 (50.0%) | 6 |
| Foreign body - Lung | 0 (0.0%) | 2 (100.0%) | 2 |
| Fracture | 22 (34.4%) | 42 (65.6%) | 64 |
| Haematuria | 0 (0.0%) | 1 (100.0%) | 1 |
| Hernia | 2 (33.3%) | 4 (66.7%) | 6 |
| Hydrocephalus | 7 (50.0%) | 7 (50.0%) | 14 |
| Intracranial infection | 5 (83.3%) | 1 (16.7%) | 6 |
| Infantile pyloric stenosis | 0 (0.0%) | 2 (100.0%) | 2 |
| Lymphadenopathy | 0 (0.0%) | 2 (100.0%) | 2 |
| Mastoiditis | 1 (33.3%) | 2 (66.7%) | 3 |
| Oral/nasal/lachrymal injury/infection | 2 (16.7%) | 10 (83.3%) | 12 |
| Osteomyelitis/arthritis | 2 (18.2%) | 9 (81.8%) | 11 |
| Penile malformation | 7 (77.8%) | 2 (22.2%) | 9 |
| Polydactyl | 2 (28.6%) | 5 (71.4%) | 7 |
| Polytrauma | 0 (0.0%) | 2 (100.0%) | 2 |
| Soft tissue infection | 9 (40.9%) | 13 (59.1%) | 22 |
| Soft tissue injury | 1 (8.3%) | 11 (91.7%) | 12 |
| Soft tissue tumour | 3 (100.0%) | 0 (0.0%) | 3 |
| Tonsilitis | 2 (50.0%) | 2 (50.0%) | 4 |
| Tracheoesophageal fistula | 1 (100.0%) | 0 (0.0%) | 1 |
| Traumatic amputation - Fingers | 1 (20.0%) | 4 (80.0%) | 5 |
| Traumatic brain/head injury | 6 (33.3%) | 12 (66.7%) | 18 |
| Upper GI bleeding | 1 (100.0%) | 0 (0.0%) | 1 |
| Ureterovesical Malformations | 1 (7.7%) | 12 (92.3%) | 13 |
| Vertebral malformation | 0 (0.0%) | 6 (100.0%) | 6 |
| Wilm's tumour | 1 (100.0%) | 0 (0.0%) | 1 |
| **Total** | **129 (34.5%)** | **245 (65.5%%)** | **374** |

Abbreviations: Pre-CP, pre-clinical pathway; Post-CP, post-clinical pathway; GI, gastrointestinal.

**Supplementary Table C**: Utilization of Access and Watch classes of antimicrobials.

|  | AWaRe Group | Pre-CP | Post-CP | p-value |
| --- | --- | --- | --- | --- |
| On admission | Access | 13 (76.5%) | 17 (85.0%) | 0.680 |
|  | Watch | 4 (23.5%) | 3 (15.0%) |  |
| After admission | Access | 112 (64.7%) | 217 (75.6%) | <0.001 |
|  | Watch | 61 (35.3%) | 70 (24.4%) |  |

Abbreviations: Pre-CP, Pre-Clinical Pathway; Post-CP, Post-Clinical Pathways.

**Supplementary Table D**: Antimicrobial utilization during pre-clinical pathway and post-clinical pathway periods, both on admission and after admission.

| Antimicrobials | AWaRe Class | On-Admission | | After admission | | Total | |
| --- | --- | --- | --- | --- | --- | --- | --- |
|  |  | **Pre-CP** | **Post-CP** | **Pre-CP** | **Post-CP** | **Pre-CP** | **Post-CP** |
| Amikacin | Access | 0 | 0 | 2 | 0 | 2 | 0 |
| Amoxicillin | Access | 3 | 1 | 11 | 23 | 14 | 24 |
| Ampicillin | Access | 0 | 1 | 0 | 3 | 0 | 4 |
| Augmentin | Access | 4 | 8 | 48 | 113 | **52** | **121** |
| Cefazolin* | Access | 0 | 0 | 1 | 13 | 1 | 13 |
| Clindamycin | Access | 0 | 0 | 1 | 3 | 1 | 3 |
| Cloxacillin | Access | 1 | 1 | 1 | 15 | 2 | 16 |
| Cotrimoxazole | Access | 0 | 0 | 13 | 7 | 13 | 7 |
| Gentamycin | Access | 1 | 1 | 4 | 0 | 5 | 1 |
| Metronidazole | Access | 4 | 5 | 31 | 40 | **35** | **45** |
| Total | | **13** | **17** | **112** | **217** | **125** | **234** |
| Cefotaxime | Watch | 1 | 1 | 36 | 33 | **37** | **34** |
| Ceftriaxone | Watch | 3 | 2 | 15 | 19 | 18 | 21 |
| Meropenem | Watch | 0 | 0 | 3 | 9 | 3 | 9 |
| Piperacillin/tazobactam | Watch | 0 | 0 | 3 | 1 | 3 | 1 |
| Vancomycin | Watch | 0 | 0 | 4 | 7 | 4 | 7 |
| Erythromycin | Watch | 0 | 0 | 0 | 1 | 0 | 1 |
| Total | | **4** | **3** | **61** | **70** | **65** | **73** |
| Amphotericin B | NA | 0 | 0 | 0 | 1 | 0 | 1 |
| Grand total | | **17** | **20** | **173** | **288** | **190** | **308** |

*Is considered Access since it is used as first line instead of Watch when it is used as second (Ref: WHO AWaRe booklet). Abbreviations: Pre-CP, Pre-Clinical Pathway; Post-CP, Post-Clinical Pathway.

**Supplementary Table E**: Appropriateness of use of Access and Watch classes of antimicrobials.

|  | AWaRe Group | | Pre-CP | Post-CP | p-value |
| --- | --- | --- | --- | --- | --- |
| On admission | Access | App | 7 (53.8%) | 16 (94.1%) | **0.025** |
|  |  | Not-App | 6 (46.2%) | 1 (5.9%) |  |
|  | Watch | App | 3 (75.0%) | 3 (100.0%) | 1.000 |
|  |  | Not-App | 1(25.0%) | 0 (0.0%) |  |
| After admission | Access | App | 47 (42.3%) | 188 (92.2%) | **<0.001** |
|  |  | Not-App | 64 (57.7%) | 16 (7.8%) |  |
|  | Watch | App | 26 (41.9%) | 80 (96.4%) | **<0.001** |
|  |  | Not-App | 36 (58.1%) | 3 (3.6%) |  |
| Overall | Access | App | 54 (43.5%) | 204 (92.3%) | **<0.001** |
|  |  | Not-App | 70 (56.5%) | 17 (7.7%) |  |
|  | Watch | App | 29 (43.9%) | 83 (96.5%) | **<0.001** |
|  |  | Not-App | 37 (56.1%) | 3 (3.5%) |  |

Abbreviations: Pre-CP, Pre-Clinical Pathway; Post-CP, Post-Clinical Pathway.

**Supplementary Table F**: Prescribers, their experience level, and antimicrobial use.

| Prescriber role | Pre-CP (n=129) | | | Post-CP (n=245) | | |
| --- | --- | --- | --- | --- | --- | --- |
|  | **App** | **Not-app** | **p-value** | **App** | **Not-app** | **p-value** |
| Medical Officer | 28 (42.4%) | 38 (57.6%) | 0.459 | 142 (91.0%) | 14 (9.0%) | 0.462 |
| Intern | 7 (33.3%) | 14 (66.7%) |  | 20 (87.0%) | 3 (13.0%) |  |
| Medical Officer | 28 (42.4%) | 38 (57.6%) | 0.087 | 142 (91.0%) | 14 (9.0%) | 0.158 |
| Specialist | 11 (26.2%) | 31 (73.8%) |  | 64 (97.0%) | 2(3.0%) |  |
| Intern | 7 (33.3%) | 14 (66.7%) | 0.554 | 20 (87.0%) | 3 (13.0%) | 0.106 |
| Specialist | 11 (26.2%) | 31 (73.8%) |  | 64 (97.0%) | 2(3.0%) |  |
| <2 Years | 9 (32.1%) | 19 (67.9%) | 0.725 | 22 (91.7%) | 2 (8.3%) | 1.000 |
| 2-5 years | 3 (25.0%) | 9 (75.0%) |  | 50 (92.6%) | 4 (7.4%) |  |
| <2 Years | 9 (32.1%) | 19 (67.9%) | 0.562 | 22 (91.7%) | 2 (8.3%) | 1.000 |
| >5 years | 34 (38.2%) | 55 (61.8%) |  | 154 (92.2%) | 13 (7.8%) |  |
| 2-5 Years | 3 (25.0%) | 9 (75.0%) | 0.528 | 50 (92.6%) | 4 (7.4%) | 1.000 |
| >5 years | 34 (38.2%) | 55 (61.8%) |  | 154 (92.2%) | 13 (7.8%) |  |

Abbreviations: Pre-CP, Pre-Clinical Pathway; Post-CP, Post-Clinical Pathway.

**Supplementary Table G** Appropriateness of antimicrobial use summarized by length of hospital stay. by

| Length of hospital stay (days) | Post-CP | | |
| --- | --- | --- | --- |
|  | App | Not-app | p-value |
| <5 days | 88 (75.2%) | 29 (24.8%) | 0.717 |
| 6-10 days | 76 (73.1%) | 28 (26.9%) |  |
| <5 days | 88 (75.2%) | 29 (24.8%) | 0.398 |
| >10 days | 108 (70.6%) | 45 (29.4%) |  |
| 6-10 days | 76 (73.1%) | 28 (26.9%) | 0.664 |
| >10 days | 108 (70.6%) | 45 (29.4%) |  |

Abbreviations: Post-CP, Post-Clinical Pathway; App, appropriate; Not-app, not appropriate.
